# Biochemical Characteristics and Potential Biomedical Applications of Hydrolyzed Carrageenans

**DOI:** 10.3390/md21050269

**Published:** 2023-04-26

**Authors:** Sanjida Humayun, Amal D. Premarathna, Vitalijs Rjabovs, Md Musa Howlader, Clarisa Naa Shormeh Darko, Il-Kyoon Mok, Rando Tuvikene

**Affiliations:** 1 School of Natural Sciences and Health, Tallinn University, Narva mnt 29, 10120 Tallinn, Estonia; 2 National Institute of Chemical Physics and Biophysics, Akadeemia tee 23, 12618 Tallinn, Estonia; 3 Institute of Technology of Organic Chemistry, Riga Technical University, P. Valdena Str. 3, LV-1048 Riga, Latvia; 4 Green-bio Research Facility Center, Institutes of Green Bio Science & Technology, Seoul National University, Pyeongchang-gun 25354, Gangwon-do, Republic of Korea

**Keywords:** autohydrolysis, polysaccharides, antioxidant, RAW264.7, HaCaT, HDF, cell proliferation, anti-inflammation

## Abstract

Seaweed contains a variety of bioactive compounds; the most abundant of them are polysaccharides, which have significant biological and chemical importance. Although algal polysaccharides, especially the sulfated polysaccharides, have great potential in the pharmaceutical, medical and cosmeceutical sectors, the large molecular size often limits their industrial applications. The current study aims to determine the bioactivities of degraded red algal polysaccharides by several in vitro experiments. The molecular weight was determined by size-exclusion chromatography (SEC), and the structure was confirmed by FTIR and NMR. In comparison to the original furcellaran, the furcellaran with lower molecular weight had higher OH scavenging activities. The reduction in molecular weight of the sulfated polysaccharides resulted in a significant decrease in anticoagulant activities. Tyrosinase inhibition improved 2.5 times for hydrolyzed furcellaran. The alamarBlue assay was used to determine the effects of different Mw of furcellaran, κ-carrageenan and ι-carrageenan on the cell viability of RAW264.7, HDF and HaCaT cell lines. It was found that hydrolyzed κ-carrageenan and ι-carrageenan enhanced cell proliferation and improved wound healing, whereas hydrolyzed furcellaran did not affect cell proliferation in any of the cell lines. Nitric oxide (NO) production decreased sequentially as the Mw of the polysaccharides decreased, which indicates that hydrolyzed κ-Carrageenan, ι-carrageenan and furcellaran have the potential to treat inflammatory disease. These findings suggested that the bioactivities of polysaccharides were highly dependent on their Mw, and the hydrolyzed carrageenans could be used in new drug development as well as cosmeceutical applications.

## 1. Introduction

In recent years, many industries such as the nutraceutical, pharmaceutical and cosmeceutical industries have increased their interest in seaweed for the development of functional food, medicine and cosmetic products due to its rich source of various bioactive compounds such as polysaccharides, pigments, fats, proteins and phenolic compounds, etc. [1]. Among all other seaweed species, red seaweeds generally contain higher-molecular-weight polysaccharides and the highest amount of sulfated polysaccharides, especially carrageenans, (30 to 70% of dry weight) which are water soluble and have a linear backbone [2]. Carrageenans are made up of disaccharide-repeating units of 1,3-linked β-d-galactose and 1,4-linked 3,6-anhydro-α-d-galactose, and based on their sulfation pattern, three major types of carrageenan are commercially available, namely κ, ɩ and λ carrageenan [3]. *Rhodophyceae* is the key class to acquiring different types of carrageenan from red algae [4]. *Kappaphycus alvarezii*, *Eucheuma spinosum* and *Furcellaria lumbricalis* are the major sources of commercial κ-carrageenan, ι-carrageenan and furcellaran [5,6,7]. Because of their unique rheology, carrageenans are frequently used in food industries as stabilizers, viscosity enhancers or gel forming agents [4,8].

Many regions in the world consume seaweeds as food, supplements or medicine due to their numerous health benefits [9]. Over the past years, marine polysaccharides, especially sulfated polysaccharides (SPs), gained great attraction for their low toxicity and various biological effects, such as immunomodulation, antiproliferation, antioxidant, anticoagulant, antiviral and antitumor activities [10,11,12,13]. In addition, some algal polysaccharides possess UV protection, anti-inflammation, antiaging, reduction-of-hyperpigmentation and wound-healing properties which can contribute to skin protection [14]. These bioactive properties of polysaccharides are closely related to their molecular weight, sulfation pattern and degree, glycosidic linkages, types of monosaccharide content, ratio of monosaccharides and so on [15].

There are several different methods including sonication [16], radiation [17] chemical [18] and enzymatic degradation [19] and autohydrolysis [20] used for the hydrolysis of polysaccharides. Trichloroacetic acid (TCA), H_2_SO_4_ and HCl are the most commonly used acids to hydrolyze polysaccharides, considering their low cost and rapid process [21]. However, low extraction yields and hydroxyl methyl make polysaccharide purification difficult [22]. Even though an enzyme is the best way to produce hydrolyzed polysaccharides due to its high substrate specificity, the production and purification of the enzyme is quite complex and expensive [23]. On the other hand, chemical hydrolysis of polysaccharides such as autohydrolysis is less complicated and involves only high temperatures and water [24]. As no chemical is used in the autohydrolysis process, it makes this process more economical and environmentally friendly, and high yields of purified hydrolyzed polysaccharides can also be ensured [25].

There are many reports that suggested the enhancement of bioactivities of algal polysaccharides after hydrolysis. A previous study suggested algal polysaccharides with lower-Mw possess higher bioactivities compared to the larger ones. Saluri et al. 2020 reported higher antioxidant properties of carrageenan with the reduction of Mw through the autohydrolysis process [20]. Enzymatic hydrolysis of the polysaccharides from *Ecklonia cava*, a brown alga, demonstrated the increased antioxidant activity as well as antiproliferation effect of the CT-26 colon cancer cell line [26]. Recently, some studies focused on the effects of algal polysaccharides on Macrophage cells. Macrophages are immune cells that activate signaling molecules such as nitric oxide (NO), interleukins (ILs) and tumor necrosis factor alpha (TNF-α) to cause inflammation [27]. Overexpression of inflammatory factors can cause severe inflammation-related diseases, including cancer, and hamper the wound-healing process [28,29]. Enzymatic hydrolysis of sulfated polysaccharides (SPs) from *Sargassum horneri* exhibited a reduction of NO production through inhibition of MAPK and NF-κB signaling pathways in lipopolysaccharide (LPS)-stimulated RAW264.7 murine macrophage cells [30].

In this study, we investigated the effect of the autohydrolysis process on carrageenans and clarified how it influences the bioactivities in relation to their sulfation patterns and Mw characteristics, as well as possible future applications of these substances.

## 2. Results and Discussion

### 2.1. Molecular Weight Analysis, Sulfate Release and Chemical Composition

**Figure 1:**
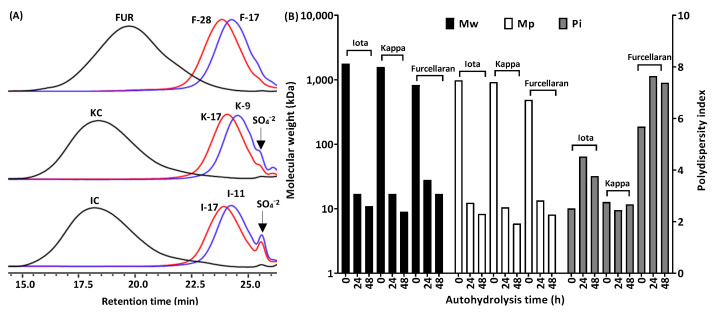
(**A**) High performance size exclusion chromatography (HP-SEC) analysis of autohydrolzed polysaccharides. Non-degraded and degraded (24 h and 48 h) furcellaran, κ-carrageenan and ι-carrageenan. (**B**) Molecular weight determination of polysaccharides. Weight average molecular weight (Mw), molecular weight of the highest peak (Mp), polydispersity index (Pi) of non-degraded and degraded polysaccharides.

Figure 1 showed the changes in the retention times of furcellaran, κ-carrageenan and ι-carrageenan after 0 h, 24 h and 48 h of autohydrolysis. Drastic changes in molecular weight were observed as the duration of autohydrolysis increased. After 24 h, furcellaran, κ-carrageenan and ι-carrageenan were 30-, 94- and 105-times smaller than their original molecular weights (Figure 1). After 48 h, furcellaran was converted to 17 kDa from 28 kDa, κ-carrageenan to 9 kDa from 17 kDa and ι-carrageenan to 11 kDa from 17 kDa.

It was confirmed that in the course of inactivation after autohydrolysis, Amberlite-Na^+^-form resin did not retain the released sulfate. During autohydrolysis, the slight release of the sulfate group was observed in ι-carrageenan and κ-carrageenan, whereas almost no sulfate was released in furcellaran. Totals of 0.007% and 0.02% of the sulfate were released from 17 kDa (K-17) and 9 kDa (K-9) κ-carrageenans after 24 h and 48 h, respectively. As for 17 kDa (I-17) and 11 kDa (I-11) ι-carrageenans, 0.03% and 0.06% of the sulfate was released. The amount of sulfate released from κ-carrageenan and ι-carrageenan during autohydrolysis is negligible, in short, it did not affect the biochemical activities of polysaccharides. Table 1 shows the total sugar and sulfate contents of the studied native furcellaran, κ-carrageenan and ι-carrageenan preparations. The measured values for the total sugar of furcellaran, κ and ι-carrageenan were 72.9%, 67.7% and 48.1%, respectively, which are in agreement with the theoretical values. Furcellaran, a hybrid carrageenan, possesses the least amount of sulfate (21%), whereas for the κ- and ι-carrageenans, the sulfate contents were 26% and 37%, respectively. No substantial changes in sulfate and sugar contents were observed during autohydrolysis.

### 2.2. Vibrational Spectroscopy

**Figure 2:**
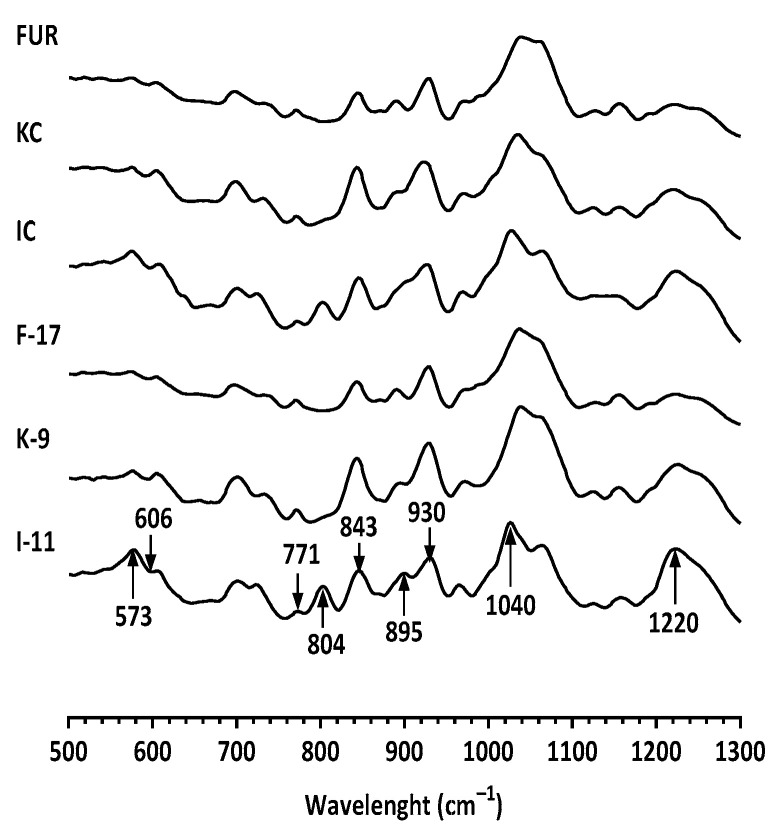
ATR-FTIR spectra of native and autohydrolyzed furcellaran, κ-carrageenan and ι-carrageenan preparations.

To determine the structural changes during autohydrolysis in furcellaran, FTIR spectra were studied (Figure 2). The highest band ranged from 1000 cm^−1^ to 1100 cm^−1^, where κ-carrageenan and furcellaran exhibited one-shouldered signals and ι-carrageenan exhibited two-separate-shouldered signals. The signals around 1000 cm^−1^ to 1100 cm^−1^ contributed to C−O, C−C, C−C−O and C−O−H vibration [31]. Both non-degraded and degraded polysaccharides had the same band at 1220 cm^–1^ which is attributed to sulfate esters, indicating sulfated polysaccharides [31,32,33].

The regions around 930 cm^−1^ and 845 cm^−1^ indicate the presence of 3,6-anhydrogalactose residues [34] and 4-linked residues of C−OH coupled with C−H [35]. ι-carrageenan spectra showed a specific signal at 804 cm^−1^ related to 3,6-anhydrogalactose-2-sulfate [34]. The spectra of the all nine polysaccharides show signals at 573 cm^−1^ and 606 cm^−1^, which raised from the bending vibration of O=S=O [36].

### 2.3. ^1^H-NMR Spectroscopy

**Figure 3:**
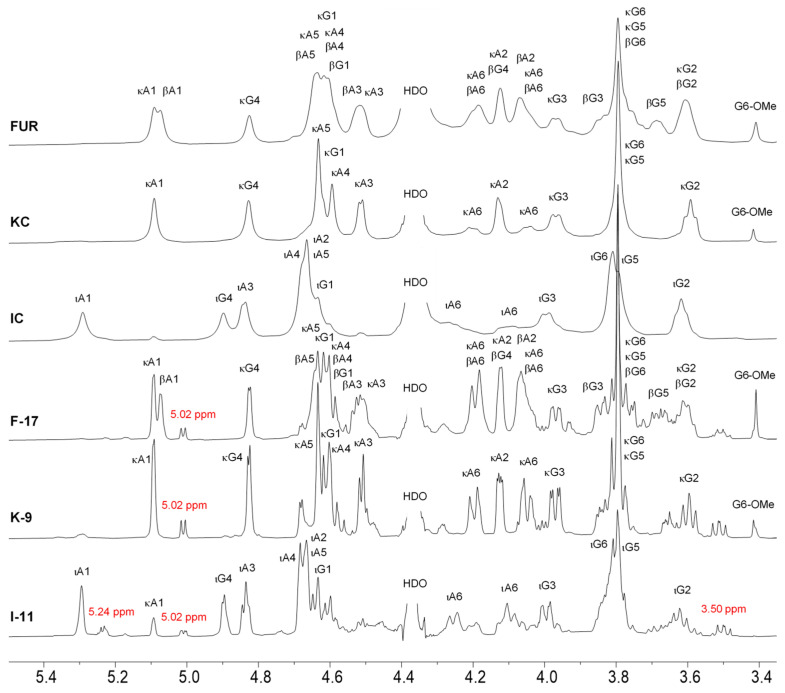
^1^H-NMR spectra of native and hydrolyzed furcellaran (FUR, F-17), κ-carrageenan (KC, K-9) and ι-carrageenan (IC, I-11) recorded at 65 °C. Signal markings refer to hydrogens attached to respective carbons from β-D-galactopyranose (G) and 3,6-anhydro-α-D-galactopyranose (A) residues from beta (β), kappa (κ) and iota carrageenan (ι) diads. New peaks arisen during autohydrolysis are indicated by red color.

Changes in a chemical structure of the polysaccharides during autohydrolysis, such as increases in the number of reducing termini via cleavage of the glycosidic linkage and loss of sulfate groups, can be determined by NMR. The ^1^H-NMR spectra of non-degraded and degraded furcellaran, κ- and ι-carrageenans are shown in Figure 3. The presence of κ-carrageenan (~8 mol% as determined by integration of the characteristic signals) in addition to ι-carrageenan is confirmed in the spectrum of native ι-carrageenan. Dyads of κ- and β-carrageenans appear in furcellaran, whereas only the κ-carrageenan dyad appears in non-hydrolyzed κ-carrageen, which indicates the higher purity degree of both polysaccharides. After autohydrolysis, a new peak appeared at 5.0 ppm in all three hydrolyzed polysaccharides. A similar peak was reported previously in κ-carrageen oligomers obtained by irradiation [17] or by hydrolysis and was attributed as a κ-carrabiose [37].

As determined by size exclusion chromatography (SEC), samples after autohydrolysis did not contain disaccharaides (neo- and carrabioses), so we assign them as a reducing terminus of the longer oligosaccharides. This signal is a major hydrolysis product of furcellaran and κ-carrageenan samples, while in the sample of ι-carrageenan, a more intensive signal at ~5.24 ppm appears. This signal can be attributed to the glycosidic proton of the reducing terminus of ι-carrageenan oligomers—the presence of a sulfate group at *O*-2 increases its chemical shift. The integral intensity of this signal is ~1.5-times greater than that of the signal at 5 ppm. At the same time, the intensities of characteristic signals of the ι- and κ-carrageenan diads have a ratio of ~75%:25%. Although increases in the intensity of the κ-carrageenan diad from ~8% to ~25% were observed, this was not due to desulfation, as no substantial release of the sulfate groups during the autohydrolysis was observed by HP-SEC analysis. In all samples, a signal at 3.5 ppm appeared. It can be attributed to a proton H2 of a reducing terminal G unit in the hydrolyzed oligomers.

**Figure 4:**
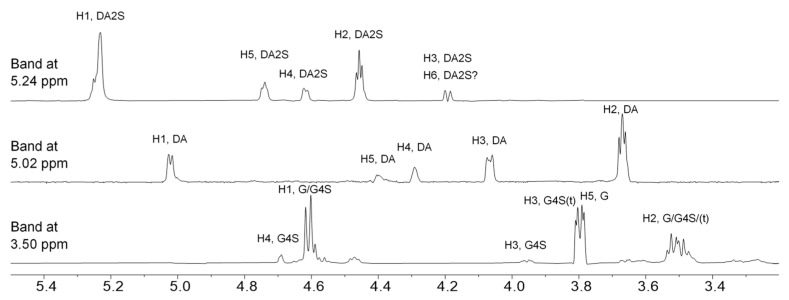
Stacked 1D TOCSY spectra of hydrolyzed ι-carrageenan samples (I-11) recorded at 65 °C. Selective excitation at designated chemical shifts was followed by spinlock irradiation during a 100 ms mixing time. Spectra show signals of protons that are present in the same carbohydrate cycle as the irradiated signal. Signal markings refer to respective protons attached to respective carbons from β-d-galactopyranose (G), β-d-galactopyranose-4-sulfate (G4S), 3,6-anhydro-α-d-galactopyranose (DA), 3,6-anhydro-α-d-galactopyranose-2-sulfate (DA2S); (t)—terminal residue from the diad lacking a glycosidic bond at *O*-3 of β-d-galactopyranose moiety.

The signal of the glycosidic proton (H1) of this galactose, as determined by selective 1D TOCSY experiments, overlaps with other major signals at ~4.6 ppm. Methoxy groups attached to G and DA residues can also be detected by ^1^H-NMR spectroscopy as signals at 3.41 (Me at *O*-6) and 3.34 (Me at *O*-2) ppm. 1D selective TOCSY (Figure 4), just like its 2D counterpart, allows for exploring a continuous CH-containing spin network, such as carbohydrate cycle. The propagation of the magnetization via a J-coupled spin system results in decreased intensity of the signals with an increasing number of bonds away from the irradiated proton. Here, a 100 ms mixing time allowed for observing at least the three closest protons. The signal at 3.5 ppm apparently represents protons belonging to different types of G units, including non- and sulfated units that may have or lack a glycosidic bond to its O-3. As a result, several lower-intensity signals appear in the corresponding 1D TOCSY. Those signals, to which the magnetization could not be transferred, can be deduced from the remaining signals in the ^1^H spectrum of the hydrolyzed ι-carrageenan based on the literature data. However, it is possible that signals of other products of the hydrolysis are not revealed by this technique and thus cannot be identified unambiguously.

**Figure 5:**
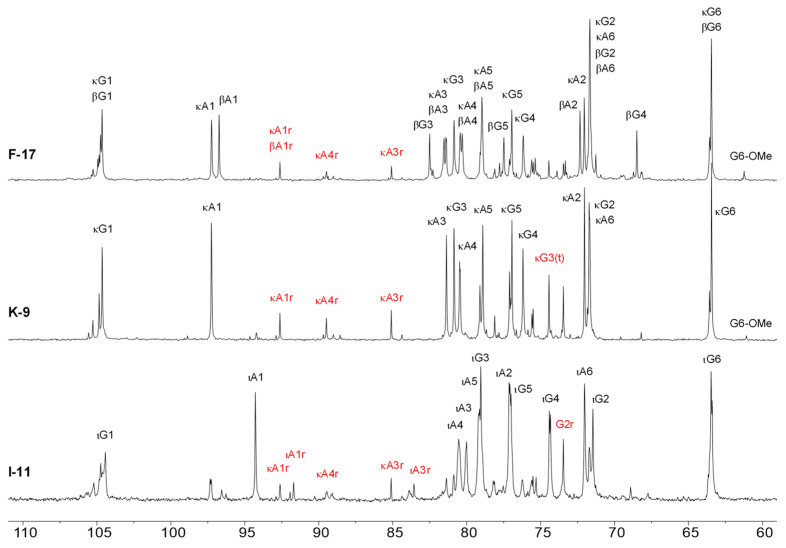
^13^C-NMR spectra of hydrolyzed forms of furcellaran (F-17), κ-carrageenan (K-9) and ι-carrageenan (I-11) recorded at 65 °C. Signal markings refer to respective carbons from β-d-galactopyranose (G) and 3,6-anhydro-α-d-galactopyranose; (A) residues from beta (β), kappa (κ) and iotcarrageenan (ι) diads; r—residue from the diad with a reducing end, (t)—terminal residue from the diad lacking a glycosidic bond at *O*-3 of β-d-galactopyranose moiety. New peaks arisen during autohydrolysis are indicated by red color.

^13^C-NMR spectra were acquired, and they exhibit major signals typical to furcellaran, κ-carrageenan and ι-carrageenan as well as lower-intensity signals that obviously belong to the reducing termini of G and DA units (Figure 5).

### 2.4. Antioxidant Activities

**Figure 6:**
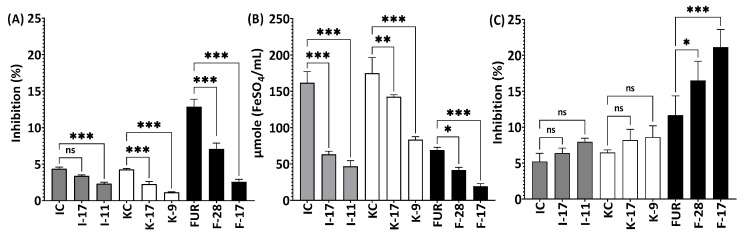
Antioxidant activities of native and autohydrolzed polysaccharides. (**A**) ABTS, (**B**) FRAP and (**C**) OH radical assay. F-28, F-17 compared with the control (FUR), K-17, K-9 compared with the control (KC) and I-17, I-11 compared with the control (IC). All experiments were performed in quadruplicate, and all data were expressed as the mean ± standard deviation (*n* = 4) and *** *p* < 0.001, ** *p* < 0.01, * *p* < 0.1 were considered statistically significant and ns means not significant.

There was a simultaneous reduction in antioxidant capacity by ABTS and FRAP during the 24 h and 48 h of autohydrolysis as Mw decreased for all three polysaccharides (Figure 6). Many studies have found a link between polysaccharide sulfation and antioxidant activity, however, in the current study the release of sulfate groups did not take major part in reduction of antioxidant capacity as during the autohydrolysis process the release of sulfate was insignificant (Figure 1, Figure 2 and Figure 4). Huang et al. (2019) showed that sulfate modification of polysaccharides enhanced free-radical scavenging activities [38]. Yang et al. (2011) investigated the antioxidant activity of native sulfated and desulfated polysaccharides, where sulfated polysaccharides showed much higher radical scavenging activity and reducing power compared to desulfated ones [39]. Similar results were shown in other studies where native sulfated polysaccharides lost their antioxidant activities after desulfation treatment [40]. As for the OH radical scavenging assay, there was no significant change observed for non-hydrolyzed and hydrolyzed κ-carrageenan and ι-carrageenan.

Interestingly, OH radical scavenging activity increased from 11.7 ± 2.7% in non-hydrolyzed furcellaran to 16.5 ± 2.7% and 21.1 ± 2.5% in 24 h and 48 h hydrolyzed furcellaran, respectively. The increased OH radical scavenging activity of furcellaran potentially correlated with its lower molecular weight and sulfate content [41]. Furthermore, polysaccharides with lower molecular weights have more reducing and non-reducing ends, which can improve OH radical scavenging activity [42]. The antioxidant activity of polysaccharides depends not only on the molecular weight and sulfate content but also on the type of monosaccharide residues, glycosidic bond and branching [42]. As several factors influence the antioxidant activities of polysaccharides and there is a lack of research on the antioxidant activity of pure polysaccharides, further study is thus required.

### 2.5. Anticoagulant Activity

**Figure 7:**
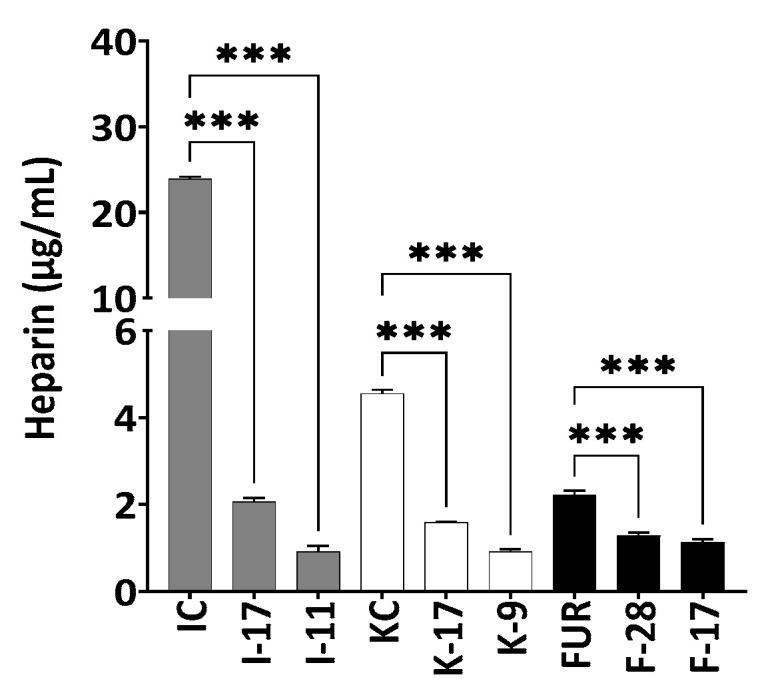
Anticoagulant activity of degraded and non-degraded furcellaran, κ-carrageenan and ι-carrageenan. 0.05% polysaccharide solution was used. All experiments were performed in quadruplicate, and all data were expressed as the mean ± standard deviation (*n* = 4) and *** *p* < 0.001 was considered statistically significant.

Among all three polysaccharides, ι-carrageenan showed the highest anticoagulant activity before autohydrolysis. Interestingly, as the incubation time of autohydrolysis increased, the molecular weight of polysaccharides decreased, as did their anticoagulant activity. The anticoagulant activity of κ-carrageenan at 1583 kDa was 4.6 ± 0.09 µg/mL (heparin equivalent), at 17 kDa was 1.6 ± 0.02 µg/mL and at 9 kDa was 0.91 ± 0.06 µg/mL. Native furcellaran (827 kDa) showed lower anticoagulant activity (2.2 ± 0.1 µg/mL) than the other two polysaccharides (Figure 7).

A continuous decline in anticoagulant activity was observed, which correlated with the Mw of the polysaccharides [43]. A significant change in the anticoagulant activity was analyzed in ι-carrageenan compared to κ-carrageenan and furcellaran during autohydrolysis. After 48 h of degradation, anticoagulant activity of ι-carrageenan was reduced by 26 times compared to non-degraded ι-carrageenan, which showed 5.2-times and 15.3-times higher reduction compared to κ-carrageenan and furcellaran, respectively. According to the previous studies, the drastic depletion of activity was not only related to the degree of sulfation, but also on Mw, which supports our current study [20,44,45,46,47]. FTIR spectra and the HP-SEC analysis confirmed that the release of a sulfate group in degraded ι-carrageenan was insignificant and it seems that for carrageenans, Mw plays even more important role in inhibition of coagulation processes compared to sulfate group localization. Liang et al. (2014) reported that algal polysaccharides with a higher number of sulfate groups possess higher anticoagulant activity [46]. Ferial et al. (2000) and Pereira et al. (2005) have shown that the position of the sulfate group and linkage patterns of sugar residues are also important for anticoagulant activity [48,49].

### 2.6. Tyrosinase Inhibition Activity

**Figure 8:**
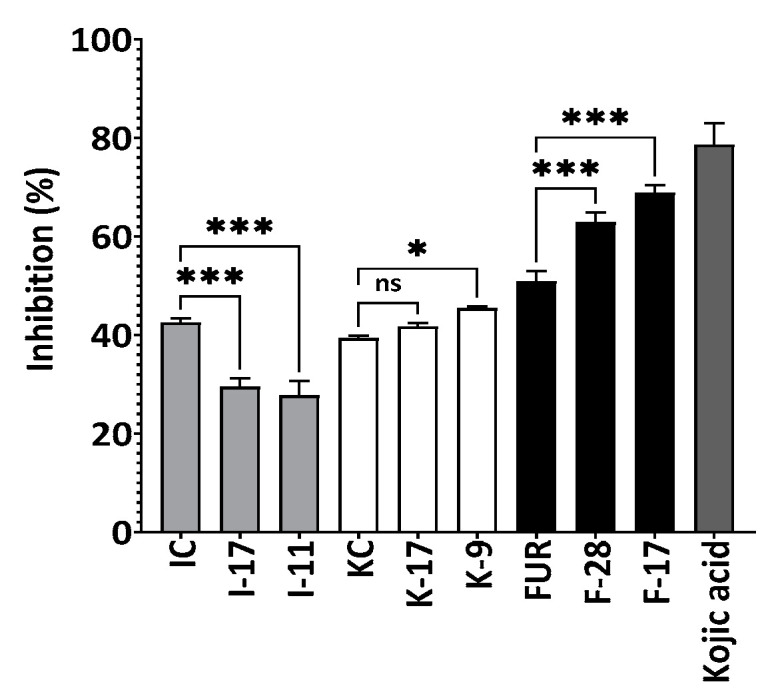
Inhibitory activity of degraded and non-degraded furcellaran, κ-carrageenan and ι-carrageenan against mushroom tyrosinase enzyme. The final polysaccharide concentration was 0.1% where kojic acid was 0.01%. All experiments were performed in quadruplicate, and all data were expressed as the mean ± standard deviation (*n* = 4) and *** *p* < 0.001, * *p* < 0.1 were considered statistically significant and ns means not significant.

Tyrosinase is a copper-containing enzyme widely found in nature (in plant and animal tissues) and is responsible for the production of melanin [50]. Excessive production of melanin can cause hyperpigmentation and melanogenesis due to these compound’s tyrosinase inhibition activity, which plays an important role in the cosmeceutical and pharmaceutical industries [51]. Figure 8 showed the mushroom tyrosinase inhibitory activities of non-degraded and degraded red algal polysaccharides. At 0.1%, the inhibitory rates for FUR, F-28, F-17 were 51 ± 2.1%, 63 ± 2% and 69 ± 1.5%, for KC, K-17, K-9 were 40 ± 0.5%, 42 ± 0.7%, 46 ± 0.4% and for IC, I-17, I-9 43 ± 0.9%, 30 ± 1.7%, 28 ± 2.9%, respectively. The data suggested that degraded carrageenans exhibit higher tyrosinase inhibition compared to native polysaccharides [52] except for ι-carrageenan. Low-molecular-weight fucoidan and the polysaccharide from *Sargassum fusiforme* inhibited tyrosinase more effectively, according to Park and Choi (2017) and Chen et al. (2016) [52,53].

As the molecular weight of furcellaran decreased, anti-tyrosinase activity increased, which can be related to the increase in antioxidant activity. A lower-Mw furcellaran demonstrated higher OH radical scavenging abilities, which can be attributed to increased antityrosinase activity because the OH group at the reducing end of the polysaccharide can form a hydrogen bond with the enzyme’s active site [54,55]. Moreover, polysaccharide binding with the tyrosinase enzyme caused a three-dimensional structural change [56] and low-molecular-weight polysaccharides might bind with the tyrosinase enzyme more firmly than high-molecular-weight polysaccharides [53]. The degree of sulfation in polysaccharides may also play an important role in tyrosinase’s inhibitory activity [57]. The inhibitory activity of degraded ι-carrageenan was reduced by 69% when compared to non-degraded ι-carrageenan, and further study is required to describe this phenomenon.

### 2.7. Cell viability of RAW264.7, HaCaT and HDF Cells

**Figure 9:**
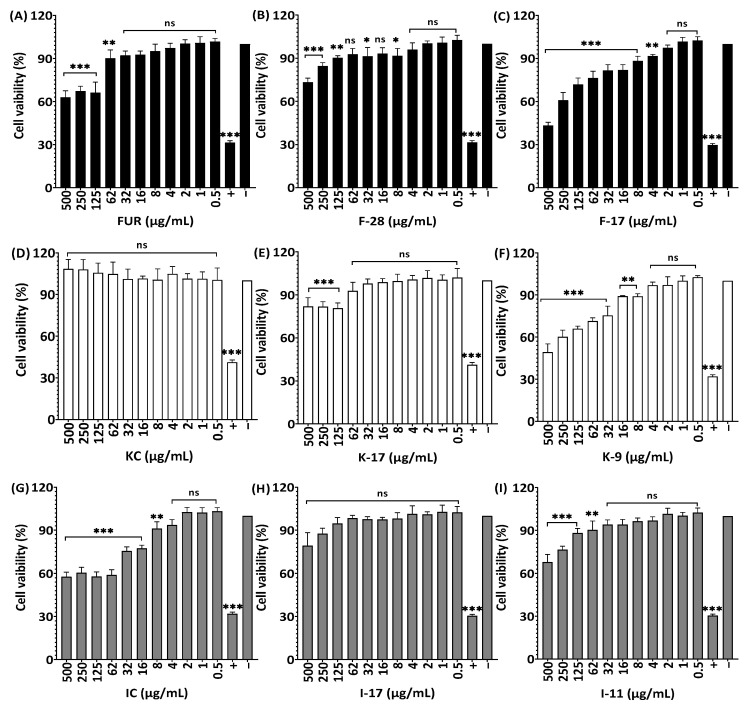
Effect of non-degraded and degraded furcellaran, κ-carrageenan and ι-carrageenan on cell viability of RAW264.7. Cells were treated with 0–500 µg/mL of FUR, F-28, F-17 (**A**–**C**), KC, K-17, K-9 (**D**–**F**) and IC, I-17, I-11 (**G**–**I**), respectively, for 24 h. All experiments were performed in quadruplicate, and all data were expressed as the mean ± standard deviation (*n* = 4) and *** *p* < 0.001, ** *p* < 0.01, * *p* < 0.1 were considered statistically significant and ns means not significant.

Based on an alamarBlue assay, it was confirmed that 2 to 0.5 µg/mL of native and autohydrolyzed furcellaran showed no influence on viability of RAW264.7 cells (Figure 9). A cell viability of 100% was observed for KC when cells were treated at 500 to 0.5 µg/mL; as for degraded κ-carrageenans K-17 and K-9, higher concentrations decreased viability, whereas lower concentrations (4 µg/mL) showed 100.6% and 97.1% cell viability, respectively. The non-degraded ι-carrageenan (IC) exhibited slighty lower cell viability compared to the degraded ι-carrageenan samples (I-17 and I-11) for RAW264.7 cells.

**Figure 10:**
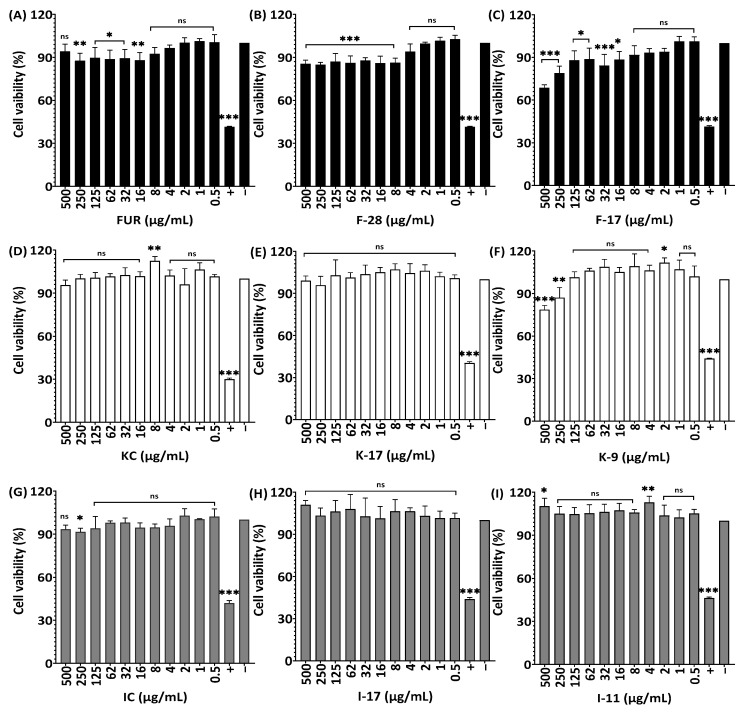
Effect of non-degraded and degraded furcellaran, κ-carrageenan and ι-carrageenan on cell viability of HaCaT. Cells were treated with 0–500 µg/mL of FUR, F-28, F-17 (**A**–**C**), KC, K-17, K-9 (**D**–**F**) and IC, I-17, I-11 (**G**–**I**), respectively, for 24 h. All experiments were performed in quadruplicate, and all data were expressed as the mean ± standard deviation (*n* = 4) and *** *p* < 0.001, ** *p* < 0.01, * *p* < 0.1 were considered statistically significant and ns means not significant.

When the HaCaT cell line was treated with 500 µg/mL and 250 µg/mL of K-9, it showed 21.4% and 12.9% less cell viability compared to KC and K-17, respectively (Figure 10D–F). At different concentrations, native and degraded ι-carrageenan showed 100% cell viability on HaCaT cells, whereas, for most degraded furcellaran and κ-carrageenan (0.5–4 g/mL), higher concentrations showed significantly lower cell viability compared with non-treated cells (Figure 10A–C,G–I).

**Figure 11:**
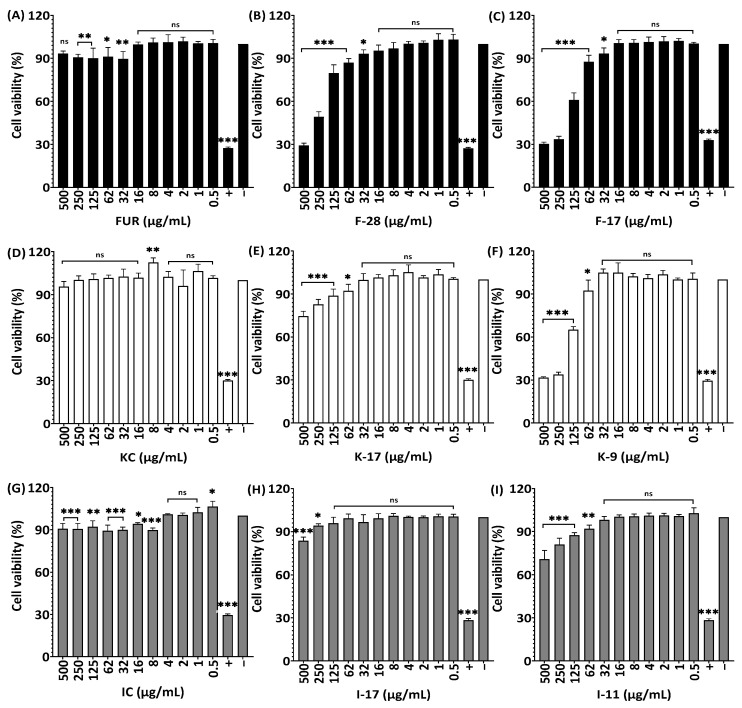
Effect of non-degraded and degraded furcellaran, κ-carrageenan and ι-carrageenan on cell viability of HDF. Cells were treated with 0–500 µg/mL of FUR, F-28, F-17 (**A**–**C**), KC, K-17, K-9 (**D**–**F**) and IC, I-17, I-11 (**G**–**I**), respectively, for 24 h. All experiments were performed in quadruplicate, and all data were expressed as the mean ± standard deviation (*n* = 4) and *** *p* < 0.001, ** *p* < 0.01, * *p* < 0.1 were considered statistically significant and ns means not significant.

The native KC sample had no significant effect on viability of HDF cells at all concentrations (0.5–500 g/mL), but as the molecular weight was reduced, the cell viability reduced by 25.5% and 68.3% compared to that of the original one (Figure 11D–F). When HDF cells were treated with (0.5–4 µg/mL) non-degraded and degraded ι-carrageenan, no effect on cell viability was observed (Figure 11G–I). Lower concentrations of furcellaran from 0.5 µg/mL to 16 µg/mL at a different Mw showed 100% cell viability (Figure 11A–C), whereas decrease in viability was observed at higher concentrations.

For cell proliferation and anti-inflammatory experiments, the amounts of furcellaran, κ-carrageenan and ι-carrageenan selected were 2 µg/mL, 4 µg/mL, 4 µg/mL for RAW264.7 cell line, 4 µg/mL, 125 µg/mL, 125 µg/mL for HaCaT cells and 16 µg/mL, 32 µg/mL, 4 µg/mL for HDF, respectively.

Based on the findings of the current study it seems that RAW264.7 cells are more sensitive to native carrageenan compared to other cell lines and viscosity can be one of the factors responsible for the apparent low cell viability. Hendriks et al. 2015 reported that viscosity greatly affects cell viability and that high viscosity could negatively influence cell viability by deforming a cell membrane [58]. It should be noted that besides the molecular structure (sulfation level) and concentration of the polysaccharide, cell viability can also be influenced by molecular weight, viscosity, the ability of the polymeric matrix to sequester certain metal ions and by the presence of non-carbohydrate impurities [46,57,58]. Further studies are highly warranted to determine the cause of the decreased cellular viability by modified polysaccharides. The highest cell viability concentrations were chosen for particular cell lines for further experiments based on the data from the cell viability assay.

### 2.8. Effects on RAW264.7, HaCaT and HDF Cell Proliferation

**Figure 12:**
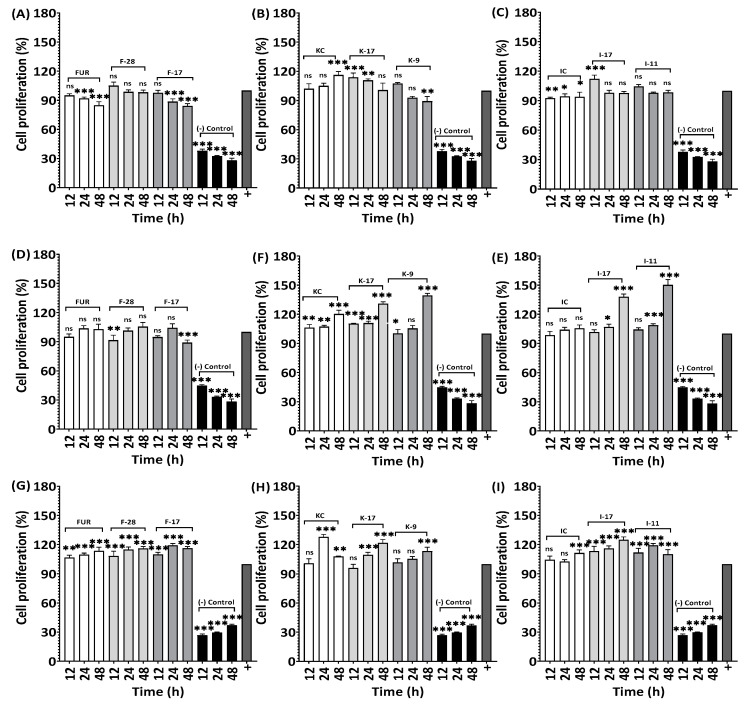
Effect of degraded and non-degraded furcellaran, κ-carrageenan and ι-carrageenan at different concentrations on proliferation ability of RAW264.7 (2 µg/mL, 4 µg/mL, 4 µg/mL polysaccharide), HaCaT (4 µg/mL, 125 µg/mL, 125 µg/mL polysaccharide) and HDF (16 µg/mL, 32 µg/mL, 4 µg/mL polysaccharide) cell lines. The cells were treated with different molecular weight polysaccharides for 48 h. (**A**–**C**) RAW264.7, (**D**–**F**) HaCaT and (**G**–**I**) HDF. All experiments were performed in quadruplicate, and all data were expressed as the mean ± standard deviation (*n* = 4) and *** *p* < 0.001, ** *p* < 0.01, * *p* < 0.1 were considered statistically significant and ns means not significant.

The WST-1 Assay Kit was used for the determination of cell proliferation assays for the non-hydrolyzed and hydrolyzed polysaccharides (Figure 12). In RAW264.7, 113.9% and 112.1% cell proliferation rates were observed after 12 h for K-17, I-17 and after 48 h, the KC showed a 116.1% cell proliferation rate. Lower-Mw κ-carrageenan (17 kDa and 9 kDa) showed 1.09- and 1.16times more cell proliferation compared to the native carrageenan, whereas hydrolyzed ι-carrageenan (17 kDa and 11 kDa) showed 1.31- and 1.42-times higher cell proliferation in contrast to the higher-Mw ι-carrageenan.

Based on this result, hydrolyzed κ-carrageenan and ι-carrageenan can be used for the development of wound healing medications, as fibroblasts and immune cells such as macrophages play an important role in wound healing [59]. Higher-Mw furcellaran (FUR) and lower-Mw furcellaran (F-28, F-17) respectively showed 113.4%, 116% and 119.1% cell proliferation for the HDF cell line, while the RAW264.7 and HaCaT cell line non-hydrolyzed and hydrolyzed furcellaran samples did not show any significant cell proliferation. Although no significant changes in cell proliferation were observed for furcellaran, lower-Mw furcellaran could be potentially used in cancer research. In 2005, Athukorala et al. reported lower-Mw polysaccharide extracted from *Ecklonia cava*, a brown alga, showed antiproliferation activity against the colon cancer cell line (CT-26) and human leukemia cell line (U-937) but didn’t affect the normal cell line [60], which showed the potential of using lower-Mw polysaccharides in cancer treatment.

### 2.9. Inhibition of Nitric Oxide Production

**Figure 13:**
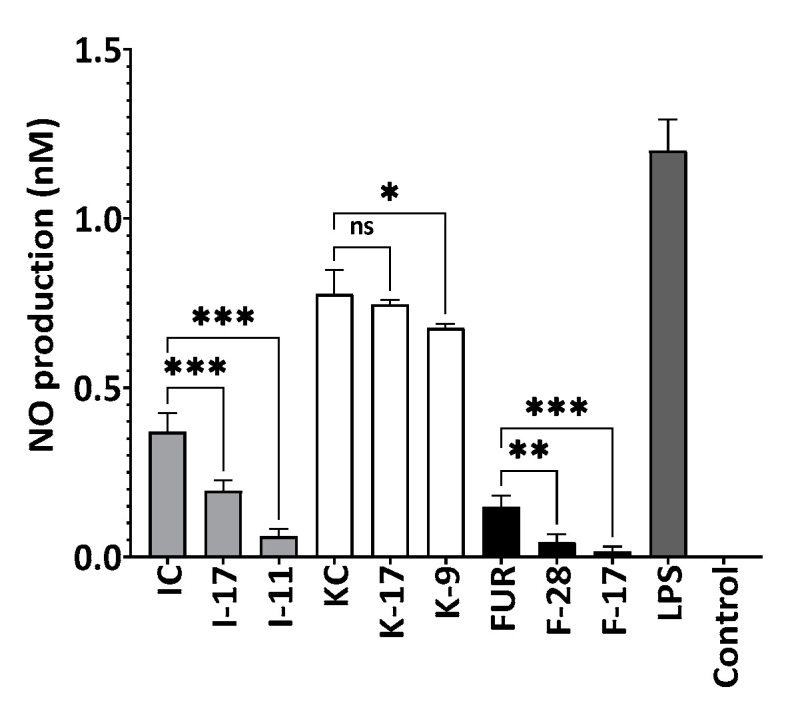
Effect of degraded and non-degraded polysaccharides on RAW264.7 macrophage cell line. F-28, F-17 compared with FUR, K-17, K-9 compared with KC and I-17, I-11 compared with IC, respectively. Cells were treated with furcellaran (2 µg/mL), κ-carrageenan (4 µg/mL), ι-carrageenan (4 µg/mL) and LPS (1 µg/mL). All experiments were performed in quadruplicate, and all data were expressed as the mean ± standard deviation (*n* = 4) and *** *p* < 0.001, ** *p* < 0.01, * *p* < 0.1 were considered statistically significant and ns means not significant.

Production of nitric oxide was investigated by lipopolysaccharides (LPS) stimulation (Figure 13), and activated RAW264.7 cells produce NO, which is an inflammatory mediator. According to Figure 13, no effect on cell viability was observed for 2 µg/mL of native and degraded polysaccharides in LPS-stimulated RAW264.7 cells. Moreover, 2 µg/mL of non-hydrolyzed and hydrolyzed polysaccharides showed a reduction in NO production. As the Mw of polysaccharides decreased, the nitric oxide inhibitory activity also increased. Among the polysaccharides, hydrolyzed furcellaran F-17 and F-28 showed 5.2- and 2.5-times higher inhibition compared to κ-carrageenan and ι-carrageenan. A significant change in NO production was observed between the original and degraded furcellaran at a concentration of 2 µg/mL. Sequentially, FUR, F-28 and F-17 furcellaran produced 0.15, 0.04 and 0.02 nM nitric oxide, respectively, showing the same pattern of OH scavenging activity. According to Wang et al., 2004, free radicals such as O_2_^−^ generate ONOO in the presence of NO, which is not only responsible for cellular damage, but can cause cell death, too [61]. Ravipati et al., 2012 and Diaz et al., 2012, also reported the correlation between antioxidant and anti-inflammatory activities [62,63].

Jiang et al., 2022 reported higher anti-inflammatory activity and lower-Mw polysaccharides through the inhibition of TLR2-dependent signaling cascades [64]. They also suggested that higher anti-inflammatory activity enhances the wound healing process [65]. Cao et al., 2014 reported that the immunomodulatory activities of polysaccharides may be affected by molecular structure rather than just Mw and sulfate content [66]. There are many papers published regarding the anti-inflammatory activities of sulfated polysaccharides and fucoidan, one of the sulfated polysaccharides, showed anti-inflammatory activities through repression of the NF-κB and MAPK pathways [67,68]. Generally, polysaccharides cannot enter the cell as a result of a higher Mw and rather interact with cell surface pattern recognition receptors (PRRs) such as TLR4 to upregulate or downregulate the genes responsible for inflammation [69,70]. As excess production of NO can cause inflammatory diseases, including cancer [71,72], non-degraded and degraded polysaccharides can be used as anti-inflammatory agents.

## 3. Materials and Methods

### 3.1. Materials

κ-carrageenan and ι-carrageenan were purchased from Tokyo Chemical Industry (TCI Co., Ltd., Tokyo, Japan). Commercial-grade furcellaran (extract from *Furcellaria lumbricalis*, Estgel 1000) was provided by Est-Agar AS (Saaremaa, Estonia). Chemicals such as 2,2′-azino-bis(3-ethylbenzothiazoline-6-sulphonic acid), ferrous sulfate heptahydrate (FeSO_4_·7H_2_O), ferric chloride (FeCl_3_·6H_2_O), sodium salicylate, sodium nitrate (NaNO_3_) potassium persulfate (K_2_S_2_O_8_), Kojic acid, β-arbutin and mushroom tyrosinase were obtained from Sigma-Aldrich (St. Louis, MO, USA). 6-hydroxy-2,5,7,8-tetramethylchromane-2-carboxylic acid (Trolox), 2,4,6-tris(2-pyridyl)-s-triazine (TPTZ), Amberlite™ IR120 H^+^-form and Na^+^-form resins were from Acros Organics (NJ, USA). Hydrogen peroxide 30% and 37% hydrochloric acid were purchased from Honeywell (Muskegon, MI, USA). Three 4-dihydroxy-l-phenylalanine (L-DOPA) were obtained from Alfa Aesar (Haverhill, MA, USA).

### 3.2. Sample Preparation

The autohydrolysis of furcellaran, κ-carrageenan and ι-carrageenan was performed according to the method described by Saluri et al. 2020, [20] with slight modification. A 0.5% solution of κ-carrageenan, ɩ-carrageenan and furcellaran was prepared, and Amberlite IR120 H^+^ form ion-exchange resin was added for acidification. After the pH reached 2.0, H^+^-form Amberlite resin was removed, and the acidic polysaccharides solution was incubated at 60 °C, 120 rpm until 48 h for continuous degradation. Autohydrolysis reaction was terminated by passing the polysaccharide solution through Amberlite Na^+^ ion-exchange column, and the obtained sample was lyophilized at −100 °C under 20 Pa (Scanvac, Brøndby, Denmark). Lyophilized polysaccharides were stored at 4 °C for further study. A small amount of the solution of autohydrolyzed polysaccharides was collected before passing through an Na^+^ ion-exchange column to confirm whether the Na^+^-form Amberlite resin retained released free sulphate ions or not.

### 3.3. Estimation of Molecular Weight and Chemical Composition

High-performance size exclusion chromatography (HP-SEC) was used to determine the molecular weight (Mw) of the polysaccharides according to Tuvikene et al., 2015 [31]. A 0.2% polysaccharide solution was prepared in MilliQ water, and for complete solubilization, was heated in boiling water. The 0.2% polysaccharide solution was diluted to 0.05% by a 0.1 M NaNO_3_ solution and filtered through a 0.22 µm RC membrane syringe filter. A total of 100 µL of the sample was injected and analyzed by size exclusion chromatography (SEC) using a Shimadzu chromatograph (Shimadzu, Kyoto, Japan) equipped with a DGU-20A5R degasser, Nexera X2 LC-30AD pump, Nexera X2 SIL-30AC autosampler, CTO-20AC column oven, RID-10A refractive index detector, Shodex OHpak SB-G (6.0 × 50 mm) guard column and two consecutive Shodex OHpak SB-806M HQ (300 × 8 mm) columns (Resonac Corporation, Tokyo, Japan). The mobile phase was composed of a single solvent system, 0.1 M NaNO_3_ in MilliQ water at a flow rate of 0.8 mL/min, and the total analysis time was 45 min. Pullulan standards ranging in size from 0.342 to 2400 kDa were used to calibrate the system for determining the peak average, weight average and number average molecular weights of polysaccharides by the LabSolutions software version 5.97 (Shimadzu, Kyoto, Japan). Release of sulfate was estimated by Na_2_SO_4_ standards, where linearity between concentrations of standards vs area was evaluated (*r*^2^ > 0.99).

The sulfate contents of the polysaccharides were determined by using the BaCl_2_-gelatin method after 0.1% furcellaran, κ-carrageenan and ι-carrageenan were hydrolyzed with 1 M HCl at 105 °C for 5 h [73]. Total sugars were measured by the phenol-H_2_SO_4_ reaction using d-galactose as a standard [74].

### 3.4. Spectroscopic Methods

#### 3.4.1. FTIR Spectroscopic Analysis

ATR-FTR spectra of the polysaccharide samples were measured by using a Nicolet iS50 FTIR spectrometer (Thermo Scientific, Waltham, MA, USA) equipped with a diamond ATR accessory, and the spectra were recorded in the 4800–380 cm^−1^ region [75].

#### 3.4.2. ^1^H-NMR Spectroscopic Analysis

The NMR spectra of the carrageenans were acquired on a DD2 500 MHz spectrometer (Agilent Technologies, Santa Clara, CA, USA) equipped with 5 mm broadband inverse (^1^H spectra) or broadband observe (^13^C spectra) probes. A 15 min temperature equilibration delay was allowed between sample insertion and NMR acquisition at 65 °C sample temperature. For ^1^H spectra, 16 scans with 25 s relaxation delay were acquired, and for ^13^C, 20,000–45,000 scans with 2.5 s recycle delay were acquired. To decrease the molecular weight, native polysaccharides were sonicated in MilliQ water for 45 min at room temperature by an ultrasonic homogenizer (Q700, 80 W) (QSONICA, Newtown, CT, USA) followed by freeze drying, while no sonication was needed for hydrolyzed polysaccharides. The 1.5% polysaccharide solutions were prepared in D_2_O (*w*/*w*) for ^1^H and 10% (*w*/*w*) for ^13^C NMR. DSS (10 mM sodium 4,4-dimethyl-4-silapentane-1-sulfonate) was used as the internal standard [31].

### 3.5. Antioxidant Assays

#### 3.5.1. Ferric-Reducing Antioxidant Power (FRAP) Assay

The antioxidant activity of polysaccharide samples was determined by FRAP assay [76] with minor modifications. The working solution was prepared by using 10 mm 2,4,6-tripyridyl-s-triazine (TPTZ) dissolved in 40 mm HCl, 300 mm sodium acetate buffer pH −3.6 and 20 mm FeCl_3_·6H_2_O mixed thoroughly in 10:1:1 (*v*/*v*/*v*) ratio. A 6 µL sample (0.2% of polysaccharide solution) or standard, 18 µL distilled water and 180 µL FRAP working solution were added and incubated at 37 °C for 30 min in dark conditions. Absorbance was measured at 593 nm by using FLUOstar OPTIMA (BMG LABTECH, San Diego, CA, USA), and results were expressed as µmole FeSO_4_/0.2% sample.

#### 3.5.2. ABTS Radical Scavenging Capacity Assay

With some modifications, ABTS radical scavenging capacity was estimated according to Wang et al., 2021 [77]. A total of 1.225 mM of potassium persulfate and 3.5 mM ABTS were prepared in 10 mL of MilliQ water. The stock solution was prepared by mixing the potassium persulfate solution and ABTS solution in 1:1 ratio and keeping it in a dark condition at room temperature for 20 h. The absorbance of the blank was adjusted to 0.700 ± 0.02 at 734 nm. A 30 µL sample (0.2% of polysaccharide solution) or standard, 120 µL ABTS working solution were added and incubated at 37 °C for 30 min in dark conditions. Absorbance was measured at 734 nm using FLUOstar OPTIMA (BMG LABTECH, San Diego, CA, USA). The radical scavenging activity was calculated as follows:

$$\mathrm{ABTS}\;\mathrm{radical}\;\mathrm{scavenging}\;\mathrm{capacity}=\left(\frac{\mathrm{Abs}\;\mathrm{of}\;\mathrm{control}-\mathrm{Abs}\;\mathrm{of}\;\mathrm{sample}}{\mathrm{Abs}\;\mathrm{of}\;\mathrm{Control}}\right)\times 100\%$$

#### 3.5.3. Hydroxyl Radical Scavenging Capacity Assay

The OH radical scavenging activities were determined to estimate the antioxidant activities with some modifications [77]. Together, 6 mM H_2_O_2_, 20 mM sodium salicylate and 1.5 mM FeSO_4_·7H_2_O solutions were prepared in MilliQ water. Polysaccharide solution 0.2% was mixed thoroughly with 6 mM H_2_O_2_, 20 mM sodium salicylate and 1.5 mM FeSO_4_·7H_2_O and reacted for 30 min at room temperature in dark conditions. The absorbance was checked at 562 nm by using FLUOstar OPTIMA (BMG LABTECH, San Diego, CA, USA). The radical scavenging activity was calculated as follows:

$$\mathrm{OH}\;\mathrm{radical}\;\mathrm{scavenging}\;\mathrm{capacity}\;\%=\left(\frac{\mathrm{Abs}\;\mathrm{of}\;\mathrm{control}-\mathrm{Abs}\;\mathrm{of}\;\mathrm{sample}}{\mathrm{Abs}\;\mathrm{of}\;\mathrm{Control}}\right)\times 100\%$$

### 3.6. Anticoagulant Activity

The effect of the autohydrolysis process on the anticoagulant activity furcellaran, κ-carrageenan and ι-carrageenan were measured as in Saluri et al., 2020, where heparin was used as a standard [20]. Degraded and non-degraded polysaccharides 0.05% were prepared in MilliQ water. To prepare the working solution, polysaccharides and human plasma were mixed in 1:9 ratios. The activated partial thromboplastin time (aPTT) test was performed at 37 °C by subsequently adding 50 μL of Erba Actime, 50 μL of sample and 50 μL of Erba CaCl_2_ and measuring it with an Erba Mannheim ECL 412 semi-automated coagulometer. The results were expressed as µg heparin/0.05% of the sample.

### 3.7. Mushroom Tyrosinase Inhibition Assay

The mushroom tyrosinase inhibition of degraded polysaccharides was determined according to Chan et al., 2011, [78] with some modifications. A total of 10 μL of tyrosinase (10 U/mL), 10 μL of sodium phosphate buffer (50 mM, pH 6.8) and 50 μL of the sample were mixed and incubated at room temperature for 10 min. After preincubation, 30 µL of l-dopa (3.3 mM) was added to the reaction and at room temperature for 30 min, the absorbance was measured once every 5 min at 475 nm using a FLUOstar OPTIMA (BMG LABTECH, San Diego, CA, USA). The final concentration of the sample was 0.1%. The percent inhibition was calculated by the following equation:

$$\%\;\mathrm{of}\;\mathrm{inhibition}=\left(\frac{\mathrm{Abs}\;\mathrm{of}\;\mathrm{control}-\mathrm{Abs}\;\mathrm{of}\;\mathrm{sample}}{\mathrm{Abs}\;\mathrm{of}\;\mathrm{control}}\right)\times 100$$

### 3.8. Cell Experiments

#### 3.8.1. Cell Culture

RAW264.7 murine macrophages (ECACC 91062702), human dermal fibroblasts (HDF, Cell Applications, Inc., San Diego, CA, USA) and human keratinocytes (HaCaT, purchased from ATCC, Manassas, Virginia, USA) were cultured in Dulbecco’s modified Eagle’s medium (DMEM, Sigma-Aldrich, St. Louis, MO, USA) containing 10% fetal bovine serum (FBS, Sigma-Aldrich, St. Louis, MO, USA), 100 U/mL penicillin and 100 µg/mL streptomycin (Pen/Strep, Thermo Fisher, Oxford, UK). All cell lines were maintained at 37 °C with a 5% CO_2_ incubator.

#### 3.8.2. Cellular Viability Test

The cell viabilities of non-degraded and degraded furcellaran, κ-carrageenan and ι-carrageenan on RAW264.7, HDF and HaCaT cells were determined by alamarBlue assay. RAW264.7, HDF and HaCaT cells were seeded on 96-well plates at 2 × 10^4^ cells/well and incubated for 12 h at 37 °C. The cells were treated with non-degraded and degraded κ-carrageenan, ɩ-carrageenan and furcellaran ranging from 0.5 to 500 µg/mL. As for the positive control (+), the cells were treated with 70% ethanol for 10 min, after which the ethanol was removed and DMEM media was added. For the negative control, DMEM media were used. After 24 h, the supernatant was removed, and cells were washed with phosphate-buffered saline (PBS). Then, 90 µL of fresh DMEM media and 10 µL of alamarBlue reagent were added to each well, and plates were incubated for 4 h in dark conditions. Fluorescence was measured at excitation 540 nm and emission 570 nm by the microplate reader FLUOstar OPTIMA (BMG LABTECH, San Diego, CA, USA). A percentage of cell viability was used to assess by comparing to the control groups.

#### 3.8.3. Cell Proliferation Assay

A density of 2 × 10^4^ cells/well of RAW264.7, HaCaT and HDF cells was seeded in a 96-well plate and incubated overnight. The amounts of degraded or native furcellaran, κ-carrageenan and ι-carrageenan used for treatment of the cells were 2 µg/mL, 4 µg/mL, 4 µg/mL for RAW264.7 cell line, 4 µg/mL, 125 µg/mL, 125 µg/mL for HaCaT cells and 16 µg/mL, 32 µg/mL, 4 µg/mL for HDF, respectively. As for the positive control (+), cells were treated with DMEM media, and for the negative control, cells were treated with 70% ethanol for 10 min. After that, the ethanol was removed and DMEM media was added. Cells were treated and incubated for 12, 24 and 48 h at 37 °C in a humidified chamber with 5% CO_2_. Cell proliferation rate was observed by the WST-1 Assay Kit (Abcam, Cambridge, UK) and performed according to the manufacturer’s instructions.

#### 3.8.4. Measurement of Nitric Oxide Production

RAW264.7 cells were seeded to 96-well plate at 1 × 10^6^ cells/well and cultured at 37 °C for 12 h. Cells were treated with 1 µg/mL of LPS, 2 µg/mL hydrolyzed or non-hydrolyzed polysaccharides and incubated at 37 °C for 24 h. Control was not treated with either LPS or polysaccharides. Nitric oxide production was determined by the Nitrite Assay Kit (Sigma-Aldrich, St. Louis, MO, USA) as per manufacturer’s instructions, and absorbance was measured at 540 nm using a microplate reader FLUOstar OPTIMA (BMG LABTECH, San Diego, CA, USA). The amount of nitrite in the sample was evaluated from a standard curve generated with a nitrite standard curve (0–10 nM in nitrite assay buffer).

### 3.9. Statistical Analysis

Experiments were conducted in quadruplicate, and the data were shown as mean ± standard error of the mean (SEM). Statistical analysis was performed using one-way ANOVA and Turkey’s post hoc multiple comparison tests on GraphPad Prism Version 9.4 for Windows (GraphPad Software, San Diego, CA, USA).

## 4. Conclusions

Although carrageenans have been extensively studied, few works have been conducted on hydrolyzed carrageenans and their bioactivities. There has been not much research performed on the cell proliferation, anti-inflammatory activities and wound healing potential of autohydrolysis-derived polysaccharides as well as the development of pharmaceutical and cosmeceutical products. Our current study reported for the first time on the inhibition of tyrosinase and NO by autohydrolysis of degraded polysaccharides. After 48 h of autohydrolysis, compared to native polysaccharides, the Mw of furcellaran, κ-carrageenan and ι-carrageenan lowered by 162.4, 175.9 and 48.7 times, respectively. During autohydrolysis, new signals arising from carrabiose oligosaccharides appeared in the NMR spectra of degraded carrageenans, indicating the cleavage of α-glycosidic linkages during the hydrolytic degradation. These oligomeric carrageenans exhibited antiproliferation and anticancer activities, which makes autohydrolysed furcellaran, κ-carrageenan and ι-carrageenan potential candidates for cancer treatment. As the Mw of carrageenan’s were reduced, the anti-inflammatory activities increased, which could be also contributed by these new signals arise during autohydrolysis. Moreover, the higher cell proliferation rate of HaCaT cells were observed for degraded κ-carrageenan and ι-carrageenan compare to the native one, which indicates their potential use as a wound healing agent. Based on these findings, we suggested degraded polysaccharides could be used for the development of the cosmeceutical and pharmaceutical industries. However, because there has been little research into the bioactivities of pure polysaccharide and their mechanisms, more in vitro (cellular and molecular) and in vivo studies are required to confirm the use of seaweed bioactivity compounds and modified polysaccharides as medical treatments.

## Figures and Tables

**Table 1:** Theoretical and measured values for the total sugar and sulfate contents of the studied native galactans.

Sample	Measured Values		Theoretical Values *		
	Total Sugar (%)	Sulfate (%)	Total Sugar (%)	Sulfate (%)	Sodium (%)
FUR	72.9 ± 1.1	21 ± 2.2	85.4 **	11.8 **	2.8 **
KC	67.7 ± 3.0	26 ± 1.6	70.9	23.5	5.6
IC	48.1 ± 3.5	37 ± 3.0	53.4	37.6	9.0

* Theoretical values are based on the idealized repeating units of the polysaccharides in their pure sodium forms. ** The theoretical value of furcellaran considers equal amounts of kappa and beta carrageenan dyads in the polysaccharide backbone.

## Data Availability

Not applicable.
